# Effect of Co-Sputtered Copper and Titanium Oxide Coatings on Bacterial Resistance and Cytocompatibility of Osteoblast Cells

**DOI:** 10.3390/nano14131148

**Published:** 2024-07-04

**Authors:** Maria P. Nikolova, Iliyan Tzvetkov, Tanya V. Dimitrova, Veronika L. Ivanova, Yordan Handzhiyski, Andreana Andreeva, Stefan Valkov, Maria Ormanova, Margarita D. Apostolova

**Affiliations:** 1 Department of Material Science and Technology, University of Ruse “Angel Kanchev”, 8 Studentska Str., 7017 Ruse, Bulgaria; itzvetkov@uni-ruse.bg; 2 Roumen Tsanev Institute of Molecular Biology, Bulgarian Academy of Sciences, Acad. G. Bonchev Str., Bl. 21, 1113 Sofia, Bulgaria; tdimitrova13@bio21.bas.bg (T.V.D.); veri55@abv.bg (V.L.I.); folie@abv.bg (Y.H.); 3 Faculty of Physics, Sofia University “St. Kliment Ohridski”, 15 Tsar Osvoboditel Blvd, 1504 Sofia, Bulgaria; 4 Institute of Electronics “Acad. Emil Djakov”, Bulgarian Academy of Sciences, 72 Tzarigradsko Chaussee, 1784 Sofia, Bulgaria; stsvalkov@gmail.com (S.V.); maria_mecheva@abv.bg (M.O.); 5 Department of Mathematics, Informatics and Natural Sciences, Technical University of Garbovo, 4 H. Dimitar Str., 5300 Gabrovo, Bulgaria

**Keywords:** titanium alloy, Cu-doped TiO_2_, copper, antibacterial coatings, *Escherichia coli*, *Staphylococcus aureus*, mechanical properties, corrosion resistance, biocompatibility, MG-63

## Abstract

One of the primary risk factors for implant failure is thought to be implant-related infections during the early healing phase. Developing coatings with cell stimulatory behaviour and bacterial adhesion control is still difficult for bone implants. This study proposes an approach for one-step deposition of biocompatible and antimicrobial Cu-doped TiO_2_ coatings via glow-discharge sputtering of a mosaic target. During the deposition, the bias of the Ti6Al4V substrates was changed. Structure examination, phase analysis, and surface morphology were carried out using X-ray diffraction (XRD) analysis, scanning electron microscopy (SEM), atomic force microscopy (AFM), and X-ray photoelectron spectroscopy (XPS). The hardness values and hydrophilic and corrosion performance were also evaluated together with cytocompatible and antibacterial examinations against *E. coli* and *S. aureus*. The results show great chemical and phase control of the bias identifying rutile, anatase, CuO, or ternary oxide phases. It was found that by increasing the substrate bias from 0 to −50 V the Cu content increased from 15.3 up to 20.7 at% while at a high bias of −100 V, the copper content reduced to 3 at%. Simultaneously, apart from the Cu^2+^ state, Cu^1+^ is also found in the biased samples. Compared with the bare alloy, the hardness, the water contact angle and corrosion resistance of the biased coatings increased. According to an assessment of in vitro cytocompatibility, all coatings were found to be nontoxic to MG-63 osteoblast cells over the time studied. Copper release and cell-surface interactions generated an antibacterial effect against *E. coli* and *S. aureus* strains. The −50 V biased coating combined the most successful results in inhibiting bacterial growth and eliciting the proper responses from osteoblastic cells because of its phase composition, electrochemical stability, hydrophilicity, improved substrate adhesion, and surface roughness. Using this novel surface modification approach, we achieved multifunctionality through controlled copper content and oxide phase composition in the sputtered films.

## 1. Introduction

Titanium and its alloys are widely used orthopaedic and dental materials for human implants because of their excellent corrosion resistance, good mechanical properties, and biocompatibility [1]. However, they demonstrate inadequate osseointegration, which is crucial to the implant’s success and the graft’s long-term stability. In contrast to orthopaedics, which bears higher mechanical requests, titanium devices used in dentistry are placed in harsher environments from a biological and chemical point of view because of changes in pH, aggressive chemicals, and proliferation of pathogens [1]. When titanium materials undergo undesirable implant-related infections at the early implantation period, it can impair osteointegration due to inflammatory processes and biofilm accumulation [2]. Bacterial colonization and the formation of biofilm on the implant surface pose a threat to the longevity of the device [3] since through biofilm bacteria elude the effects of immune responses and drugs. Commercially available implants confirmed improved osseointegration and success rates by tuning the topography and surface chemistry [4] but none of those currently existing in the market have proven antimicrobial properties for clinical use [5]. Depending on the severity of the fracture, the risk of infection during orthopaedic replacement ranges from 0.4% to 16.1% [6]. The main pathogenic species among orthopaedic isolates of implant-associated infections are *Staphylococcus aureus* (34%), *Staphylococcus epidermidis*, *Pseudomonas* (8%), *Enterococcus* (5%), *Escherichia* (2%), and others [7]. A revision surgery because of periprosthetic joint infection is a complication that leads to significant financial repercussions [8]. Therefore, designing implants with specific biology-related chemical and physical surface properties with combined cell stimulatory capacity and antibacterial potential for hard implant applications is a feasible but challenging strategy.

Coating technologies have been applied to create bioactive surfaces similar to the bone in terms of composition and topography, thus stimulating the cellular response and growth of new bone around the implant surface. Surface coatings for hard implant applications can be achieved by various chemical, physical, and mechanical techniques or combinations of these. Though each technique has its own merits and shortcomings, effective technologies that allow precise control over the process parameters are physical vapour deposition (PVD) processes. The formation and growth of sputtered thin films are highly dependent on the gas discharge parameters and energy fluxes to the substrates [9]. Furthermore, owing to the high energy of the ionized particles in the plasma, the coatings have strong bonding to the substrate and favourable nanostructured surface for implant bonding and new bone formation [10,11]. The sputtering can accommodate simple reactions as well as prepare oxides by introducing oxygen gas in the sputtering chamber. At the same time, dual sputtering sources can be used for the deposition of multi-component films [12]. 

Titania (TiO_2_) coatings have recently received widespread attention in the biomedical field because of their excellent corrosion resistance and biocompatibility [13]. It was found that by controlling TiO_2_ hydrophobicity, the protein adhesion or anti-fouling capabilities of surfaces can be altered [14]. Various PVD methods like cathodic arc evaporation, magnetron sputtering, glow discharge sputtering, electron beam evaporation, pulsed laser deposition, and thermal evaporation have been used for the deposition of TiO_2_ films [15,16]. In a previous study, we demonstrated that glow-discharge-sputtered TiO_2_ deposited on the TiN interlayer yielded improved surface characteristics, adhesion, and viability of osteogenic MG-63 cells compared with the bare implant alloy [16]. Moreover, the good mechanical properties and adhesion of sputtered films make their physiological performance suitable for load-bearing components that undergo loading forces such as those of biting, chewing, or speaking. Thus, minimal risk of friction-induced debris or corrosion occurs, which enhances the reliability of the coated devices. 

To improve the cellular interaction of coatings, PVD processes can be coupled with chemical etching or electro-physical processes for roughening of the substrate that creates a particular pattern on the implant material. However, due to increased surface area, rougher implant surfaces boost the risk of microbial adhesion and therefore require additional surface modification to reduce bacterial load [17]. An optimal biomaterial surface should prevent bacterial adhesion during the initial stages of infection and inhibit subsequent microbial growth at a later stage [18]. The standard strategy of employment of antibiotics to treat infections around the implant suffers from difficulty in reaching effective inhibitory concentrations around the tissue through blood circulation, drug resistance, and risk of side effects [19]. Inorganic particles and ions are promising candidates because of their high stability, efficacy at low concentrations [20], improvement of the mechanical performance of bone grafts [21,22], low costs, and reduced side effects as opposed to growth factors or genetic engineering approaches [23]. Compared to common antibacterial agents like Ag and Zn, copper (Cu) is an essential trace element that can deliver optimum antimicrobial properties. Its multiple oxidation states (Cu, Cu^+^, and Cu^2+^) demonstrated effective antimicrobial efficacy and cell compatibility in scaffolds [21,22,23]. Different studies have shown that Cu-containing TiO_2_ surfaces possess anti-fouling properties and antibacterial activities because of copper ion release and ROS generation [24], interference with DNA replication, and disruption of cell membranes [25]. It is believed that the negatively charged cell surface of Gram-positive bacteria readily absorbs positively charged ions electrostatically because of the presence of a peptidoglycan layer (20–80 nm) [26]. However, the antimicrobial effect of Cu ions was found to be time-dependent, requiring a longer period to kill bacteria at a safe dose [27]. Therefore, it is considered that particles containing Cu inhibit the adhesion and proliferation of bacteria employing contacting sterilization [28].

Simultaneously, Cu takes part in many enzyme-based processes for bone metabolism and cross-linking of bone elastin and collagen [2]. Cu itself is also angiogenic, stimulating new vessel formation due to the abundance of copper-binding proteins in serum [29]. Additionally, Cu^2+^ released from the surface could act as an inflammation-modulatory agent that activates macrophages to engulf and kill bacteria and enhance osteointegration [30]. Given all that, introducing Cu into a bioactive coating at a suitable concentration can give the implant surface improved osteogenesis capability without reducing biocompatibility and bactericidal properties. However, excess copper concentration beyond a certain threshold can trigger cell toxicity. For example, Zhang et al. reported that Cu-doped TiO_2_ coatings can inhibit the adhesion and proliferation of fibroblast cells at a concentration equal to 1.93 wt% [31]. These facts underline the necessity to find and maintain a balance between the positive effectiveness and cytotoxicity of Cu within the coating. If the surface modification has steady and durable antibacterial properties beneficial for avoiding or lowering bacterial properties without compromising the surface bioactivity, it will have a high added value. 

In the literature, there are plenty of studies demonstrating that by doping the electrolyte with a copper source, Cu-containing TiO_2_ coatings produced by micro-arc oxidation (MAO) or plasma electrolytic oxidation (PEO) can effectively increase cytocompatibility and angiogenesis while modulating the inflammatory response and minimising bacterial adhesion [24,31,32,33,34,35]. However, fewer studies focus on the sputtering technologies to obtain Cu-doped TiO_2_ coatings with high purity. For example, He et al. obtained intermetallic CuTi films by magnetron sputtering that were subsequently annealed in air to form CuO-doped TiO_2_ coatings [36]. A similar approach was applied by Stranak and his colleagues and by Lu et al. [12,37]. However, as far as we know, none of them used a one-step PVD process to obtain copper-containing TiO_2_ films by co-sputtering Ti and Cu from mosaic target material in an oxygen atmosphere. The objective of this work is to assess new biomedical Cu–TiO_2_ coatings deposited on a Ti6Al4V alloy via one-step glow-discharge sputtering at variable bias voltages (0, −50, and −100 V) in order to integrate both challenges, namely antibacterial activity with regenerative characteristics, using a cost-effective approach. SEM, XRD, and XPS techniques were used to identify the coatings’ structure and composition. The coatings’ mechanical endurance, wettability, and surface roughness were assessed through scratch and nanoindentation tests, contact angle measurements and AFM, in that order. Both Gram-positive (*S. aureus*) and Gram-negative (*E.coli*) strains were used to test the sensitivity of bacteria, whereas MG-63 osteoblastic cells were utilized to evaluate the in vitro physiological effects of the coatings.

## 2. Materials and Methods

### 2.1. Sample Preparation

Ti6Al4V (Gr 5) alloy with a composition of 6.23 wt% Al, 4.18 wt% V, 0.12 wt% Fe, 0.17 wt% O, 0.014 wt%, H, and Ti, was used for the experiment. Samples with a dimension of 14 × 14 × 2 mm were laser cut and subjected to acid etching in HCl (PanReac AppliChem, Heilbronn, Germany) acid for exposure times of 4 h (at 60 °C). After receiving an additional dH_2_O wash, 96% ethanol was used to clear the surface of each sample.

A П-shaped sputtering device in a cubic vacuum chamber with water-cooled walls was utilized for the glow discharge deposition. Mixed Ti–Cu co-sputtered material was assured by placing Cu (purity 99.98 at%, Ø2 cross section) on the surface of Ti (purity 99.95 at%, 6 mm thickness), thus obtaining mosaic target material with inserted 58 pure Cu wires on the top etching area [38]. The ratio of the components in the whole electrode was estimated to be equal to 418:1 = Ti:Cu. The sputtering chamber was evacuated to a base pressure of 3 × 10^−1^ Pa. To improve the adhesion of the oxide film, the samples were bombarded by a glow discharge for 15 min in a pure argon atmosphere at a working pressure of 8 × 10^0^ Pa and a target voltage of 960 V (5 A current). Then, CuO/TiO_2_ coating deposition took place in a pure O_2_ atmosphere at a working pressure of 8 × 10^0^ Pa and a target voltage of 1020 V (4 A current) for a deposition time of 240 min. For each set of samples, the substrate bias was equal to 0 V (grounded), −50, and −100 V at a constant distance of 85 mm from the cathode. The aforementioned technological settings were chosen after extensive testing and were thought to be the most appropriate. By adjusting the Ti:Cu ratio, target voltage, and other process parameters, a composite coating with the right biological response and good stoichiometry was formed. As a result, we chose them to investigate how the Cu-TiO_2_ coatings’ biological characteristics and structure were affected by the applied negative bias voltage.

Polished Ti6Al4V samples for adhesion evaluation and pure Ti foils for cross-section observations were also coated during the processes.

### 2.2. Characterization

The implant surface morphology and composition were examined with a scanning electron microscope (SEM, LYRA I XMU, Tescan, Brno, Czechia) equipped with an energy dispersive spectrometer (EDS, Quantax 200, Bruker, Billerica, MA, USA). The phase composition was analyzed using X-ray diffraction (XRD, URD-6 Seiferd&Co, Antweiler, Germany) within 2θ range of 20–80° in a step of 0.1°, Ni-filtered CuK_α_ radiation (λ = 0.154178 nm), and a symmetrical Bragg-Brentano mode. The scanning rate was 6 s per step.

The topographic images were measured with MFP—3D Classic AFM (Asylum Research, Oxford Instruments company, Abingdon, UK). The measurements were taken in AC Air Topography mode (tapping mode) with a conventional tapping mode cantilever suitable for measuring thin films and coatings. The scanned area was equal to 10 × 10 μm. The results are averaged from 5 independent measurements. Data are presented as mean ± standard deviation.

Kratos Analytical Ltd.’s AXIS Supra electron spectrometer (Stretford, UK), which has an analysis chamber with a base pressure better than 10^−7^ Pa, was employed to perform X-ray photoelectron measurements. The AlK_α_ non-monochromated X-ray source (1486.6 eV) was used for the measurements using a 0.3 × 0.7 mm beam spot. Since a charge compensator was used, no energy calibration was done. The full width at half maximum (FWHM) of the Ag3d_5/2_ photoelectron peak indicates that the instrumental resolution was better than 0.5 eV. The recorded spectra had an instrumental resolution of ±0.1 eV overall. Data analysis was performed using CasaXPS Version 2.3.26PR1.0 software (Casa Software Ltd., Teignmouth, UK). A Shirley-type background was subtracted as part of spectral processing [39]. Based on the typical spectra of iron and cobalt for various oxidation states, a symmetrical Gaussian–Lorentzian curve fitting is used to get the peak positions and areas. Using the Scofield method to normalize peak areas to photoionization cross-sections, the relative concentrations of several chemical species were determined [40].

Hysitron TI 980 instrument (Bruker, Billerica, MA, USA) in quasi-static nanoindentation tests were utilized to determine the nanohardness of the coated samples. A basic Berkovich indenter with a camber radius of about 150 nm was utilized. A standard specimen of fused quartz with a known elastic modulus (69.6 GPa) was employed for tip-area calibration. The nanoindentation tests were conducted under load control mode, applying a peak force of 10 mN. To collect statistical data and quantify the dispersion of the load-displacement curves due to surface roughness, 49 indents (7 × 7; 10 µm) were done in each test. Using an optical microscope and a conventional Rockwell-C diamond indenter, a CSEM-Scratch tester was employed to analyse the adherence of coatings up to a normal load of 30 N.

Measurement of the static contact angle employed the sessile drop method. The samples were cleaned with ethanol and heated at 50 °C for 30 min to remove all possible volatile organic residues. Then, three drops (5 μL) of distilled water were consecutively put on the surface of each sample to report reliable average values. A series of images of the three drops on each surface were captured at constant time intervals for 300 s at room temperature. At the end of the sequence, advancing contact angles (±standard deviation values) were obtained. The contact angle values were measured from photos using Autodesk AutoCAD 2016.

Electrochemical tests were carried out in SBF solution using potentiostat/galvanostat Interface 1010E (Gamry Instruments, Warminster, PA, USA). The measurements were conducted in a three-electrode cell—a working electrode with 1 cm^2^ exposed area in SBF solution, a platinum wire as a counter electrode and Ag/AgCl as a reference electrode. The SBF solution was prepared by sequential dissolving of NaCl, NaHCO_3_, KCl, K_2_HPO_4_.3H_2_O, MgCl_2_.6H_2_O, CaCl_2_, and Na_2_SO_4_ in distilled water and buffered to pH 7.4 with tris hydroxymethyl-aminomethane ((CH_2_OH)_3_CNH_2_)) and 1 N HCl at 37 °C according to [41]. The ion concentration in the solution was almost equal to that in human blood plasma. Before the potentiodynamic measurements, the samples were immersed in naturally aerated SBF (80 mL) at 37 °C for 1800 s to establish balanced open circuit potential. The potentiodynamic polarization curves were measured with the initial potential of −250 mV and the final potential of 750 mV vs. OPC at a speed of 0.167 V/s. Tafel linear extrapolation was used to estimate the corrosion current density (i_corr_) and corrosion potential (E_corr_). 

The copper released from the TiO_2_-Cu films deposited on the Ti6Al4V substrate was measured using flame atomic absorption spectrometry (PerkinElmer AAnalyst 400, PerkinElmer, Inc., Waltham, MA, USA) in an air–acetylene flame, under optimal instrumental parameters ensuring maximum signal-to-noise ratio. Four hundred microliters of 0.9% NaCl solution prepared in high-purity water (Millipore Corp., Milford, MA, USA) was added to the surface of each group of coatings (three independent technical preparations in duplicates) and incubated at 37 °C. Ion release was measured at 5, 24, and 48 h for each sample per coating method.

### 2.3. Cytocompatibility Evaluation

Using human osteosarcoma cells (MG-63, CRL-1427), the impact of sample surface modification on cell adhesion, proliferation, and growth was evaluated. The cells were maintained in High Glucose Dulbecco’s Modified Eagle Medium (DMEM, Gibco, Life Technologies Limited, Paisley, UK) containing 10% foetal bovine serum (FBS, Gibco), 100 units/mL penicillin, and 100 µg/mL streptomycin in a humidified CO_2_ atmosphere at 37 °C. They were routinely checked for mycoplasma contamination by 4,6-Diamidin-2-phenylindol staining (DAPI, Sigma-Aldrich, St. Louis, MI, USA) and were found free of it. 

A minimum of three samples from each group (non-coated, 0 V, −50 V, and −100 V coated) were examined for bacterial contamination prior to all cell studies. If contamination was not detected, we ran more tests using the series.

In all experiments, 12-well plates were used, and the cells were seeded on the sample surface (14 × 14 mm) at 6.0 × 10^4^ cells/cm^2^ density in a complete DMEM medium. The cell adhesion experiment involved plating MG-63 cells on the substrates for three hours. Subsequently, nonadherent cells were removed by washing, and the adherent ones were detached by trypsinization and counted with an automated cell counter (Countess™ 3 Automated Cell Counter, Invitrogen, Waltham, MA, USA). To determine the percentage of cell adhesion, the cell number count at 3 h was divided by the initial cell number plated and multiplied by 100.

For cell growth, 2.0 mL of growth media was added per well after 3 h and further incubated for 24, 48, and 72 h. Cell growth was determined using MTT assays [36,37,38,39,40,41,42]. Briefly, 200 µL of MTT solution (5 mg/mL) was added to each well, MG-63 cells were grown for up to 72 h and then further incubated for 3 h at 37 °C. To dissolve the formazan product of the MTT, cell media was removed and 300 µL/surface of 100% anhydrous isopropanol was added. After the formazan was fully extracted, the samples were taken out and a DTX 880 spectrophotometer (Beckman Coulter, Inc, Wals, Austria) was used to determine the optical density of the resulting solutions at 550 nm. Three independent technical experiments in duplicates were performed for each experiment. 

In the cell morphology assay, after sterilizing surfaces overnight in 96% ethanol, the MG-63 cells were seeded onto each specimen at a density of 3.0 × 10^4^ cells and cultured for 24 h. The samples were then gently rinsed 2 times with Versene solution (ThermoFisher Scientific) and fixed with 3.7% methanol-free paraformaldehyde (ThermoFisher Scientific) in PBS. The cytoskeleton protein F-actin was stained with Alexa Fluor 488 Phalloidin (Invitrogen) for 30 min. Following three washes with PBS and two with water, the slides were mounted in UltraCruz fluorescence mounting medium (Santa Cruz Biotechnology, Dallas, TX, USA) and images taken with a Zeiss Axiovert 200 M fluorescence microscope.

### 2.4. Antibacterial Evaluation

The antibacterial properties of the coatings were revealed using the plate counting method. *Escherichia coli* (*E. coli*) K12 AB1157 (F- thr-1 leu-6 proA2 his-4 argE3 thi-1 lacY1 galK2 ara-14 xyl-5 mtl-1 tsx-33 rspL31 supE44) strain was purchased from the National Bank for Industrial Microorganisms and Cell Cultures (Sofia, Bulgaria) and *Staphylococcus aureus* 6538 P was purchased from ATCC (USA). Single *E. coli* and *S. aureus* colonies were inoculated into 5 mL of sterile Lysogeny broth (LB) medium containing 1% protein hydrolysate, 0.5% yeast extract, and 0.5% NaCl, adjusted to pH 7.4. Cells were cultivated overnight at 37 °C. The next day, 100 μL of these cells were placed in 10 mL of new sterile LB medium. The cells were cultured at 37 °C to 0.6 OD (optical density). Then, 1 mL of the cells were centrifuged for 5 min at 2500 rpm. The supernatant was discarded and the bacterial pellet dissolved in 1 mL of sterile PBS. A 100 μL volume of bacterial suspensions was dropped on the coated and etched Ti6Al4V alloy, flame-sterilized (3 s), and incubated at 37 °C for 24 h. After incubation, aliquots of 10 μL were taken and diluted 100,000 times for *E. coli* and 10,000 times for *S. aureus*. A 100 μL volume of each dilution was seeded on LB agar plates. After 24 h of incubation at 37 °C, the CFU were photographed and counted. Formula (1) was used to obtain the inhibition percentage:

R = (B − A)/B × 100, %(1)

where A and B are the colony numbers for the test and control samples, respectively.

### 2.5. Biofilm Formation

The LIVE/DEAD BacLight Bacterial Viability Kit (Invitrogen Inc., Carlsbad, CA, USA) was used to analyse the biofilm formation and the viability of the adhered bacteria on the surfaces. It has two stains: SYTO-9-labelled live cells in green and propidium iodide (PI)-labelled dead cells in red. Before staining, an *S. aureus* suspension prepared as above was grown on all surfaces for 24 h. Following 3 washes with PBS, the biofilms formed were stained following the manufacturer’s instructions. Afterwards, the samples were mounted in UltraCruz fluorescence mounting medium (Santa Cruz Biotechnology, USA) and observed under a Zeiss Axiovert 200 M fluorescence microscope.

### 2.6. Statistical Analysis

All data are expressed as mean ± standard deviations of at least three independent technical experiments conducted in duplicate. The data were evaluated using analysis of variance (ANOVA) followed by Tukey’s post-hock test. Differences in the results at *p* < 0.05 were considered statistically significant. The statistical analyses were carried out using the PASW 18.0 statistical software package (IBM) for Windows.

## 3. Results and Discussions

**Figure 1:**
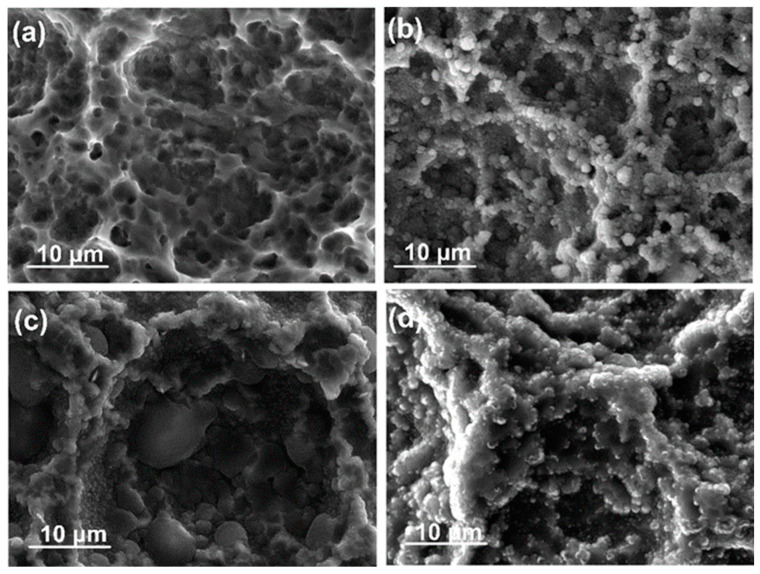
Representative SEM images of the (**a**) etched alloy surface and (**b**) TiO_2_/CuO coated samples at 0 V, (**c**) at −50 V, and (**d**) at −100 V bias voltages.

The surface topography of the etched Ti6Al4V alloy is presented in Figure 1a. The alloy yields a rather rough surface with macro-pits of different sizes. The micro-texture of the surface demonstrated small depressions and prominences resembling those of spongy bone structure. 

**Figure 2:**
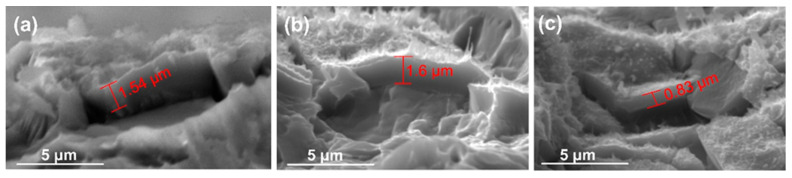
Representative cross-section images showing the thickness of the coatings obtained at (**a**) no bias, (**b**) −50 V bias voltage, and (**c**) −100 V bias voltage.

The coating morphology is slightly changed under the influence of the bias voltage (Figure 1b–d). There is no obvious effect on the spherical shapes at the top of the grains and aggregate size. All coatings seem compact and uniform, containing nano-fine grains. The coating thickness is affected by the change in bias voltage (Figure 2). When the bias voltage is increased from 0 to −50 V, the thickness rises slightly from 1.52 ± 0.06 μm to 1.61 ± 0.09 μm because the adsorption and chemical reactions kinetics are accelerated by the neutral groups and positive ions in the plasma migrating to the surface. For the biased sample with a voltage of −100 V, the film thickness reduces to 0.84 ± 0.07 μm, probably because of the densification of the microstructure and the “self-sputtering” effect, which intensifies with the increase in bias [43]. However, the typical columnar morphology of the sputtered Cu-doped TiO_2_ thin films is not changed. 

**Figure 3:**
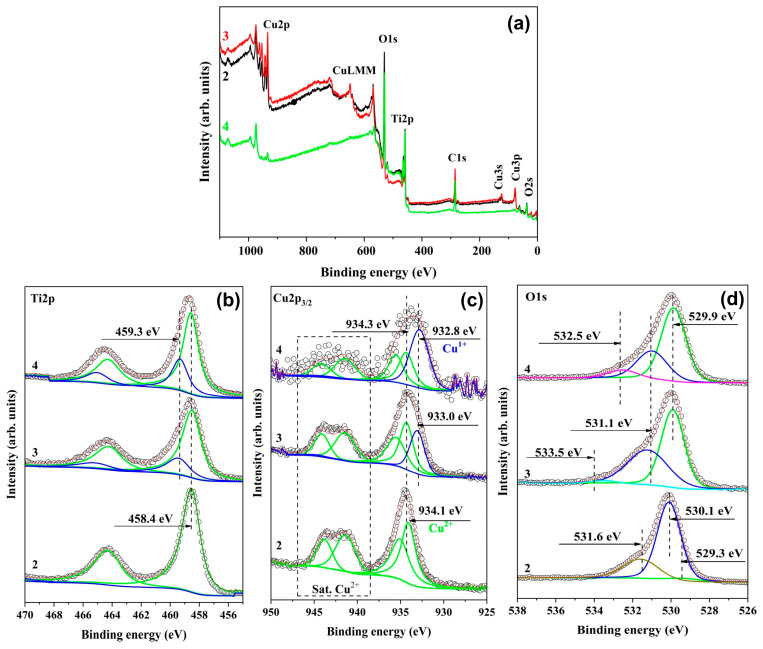
XPS survey scan of the coatings (**a**) and core level binding energy spectra of Ti 2p (**b**) Cu 2p (**c**), and O 1s (**d**). The spectra marked with 2 indicate 0 V biased, 3 indicate –50 V biased, and 4 indicate –100 V biased coatings.

The chemical state and composition of the topmost surface of the PVD films were determined via XPS analysis. Figure 3a shows the survey scan spectra of the three coatings where only Cu, Ti, O, and C are found, with no evidence of the presence of other elements. The C1s peak at 285.0 eV can be attributed to C-C or C-H bond formations from the residual gases in the vacuum system and contamination of the sample surface.

The high resolution of the Ti 2p spectrum in 0 V biased samples (Figure 3b) at 458.4 and 464.1 depicts the common binding energies of Ti^4+^ in TiO_2_, with a separation of 5.7 eV [44]. The Ti 2p peak appearance (broadening) of the −50 V and −100 V biased samples may suggest highly dispersed Ti^4+^ ions or nanocrystalline oxide formation and/or participation of titanium in an intermetallic oxide compound. Similar to the phenomenon observed in TiO_2_/HfO_2_ thin films by Ismail et al. [45], the peaks at higher binding energies (459.3 eV and 465 eV) might be attributed to the Ti amalgamation, which could change the bonding characteristics of Ti atoms due to the development of the ternary oxide phase such as CuTiO_x_. The authors of ref. [46] explained this shift by a rise in the electron density of Ti-O bonds in the TiCuO_x_ due to variations in the electronegativity of Ti, Cu, and O. It is important to note that no hint of Ti^3+^ or Ti^2+^ oxidation states is visible in the Ti 2p peaks of any sample.

For the non-biased sample, Cu 2p of Cu 2p_3/2_ centred at 934.1 eV together with the doublet shake-up satellite peaks of Cu 2p at about 941.2 and 943.5 eV (Figure 3c) is assigned to the Cu^2+^ oxidation state [47] within the oxide coating. The intensity of the satellite reduces with the increase in bias. The broader Cu 2p peaks in the −50 V biased sample with still-pronounced double satellite peak at about 9 eV higher binding energy (BE) than the main Cu 2p_2/3_ maximum confirms the presence of CuO (formally 3d^9^) since this satellite is absent for only the Cu_2_O phase due to their filled 3d shell [48]. Both Cu^2+^ and Cu^1+^ phases have formed on the −50 V film surface since the signal detected at 933 eV is assigned to the Cu^1+^ state. However, both BE values seem to increase from what is reported on the NIST database for CuO (933.6 eV) [49] and Cu_2_O (932.4 eV) [50], suggesting that copper oxide phases are not fully stoichiometric at the surface. The Cu 2p peaks of the −100 V biased sample indicate a similar position as those of the −50 V biased specimen but with a predominant lower valence state. Copper-substituted TiO_2_ structure [51] and the formation of CuTiO_x_ phases are likely the cause of the co-existence of Cu with higher and lower valence states. The peak at 934.8 eV corresponds to Cu(OH)_2_ adherent to the coating surface. 

The O1s XPS spectra vary in several ways as a result of bias variations (Figure 3d). The oxygen peak in the non-biased sample is far more symmetrically associated with the reduced surface whereas those of −50 and −100 V biased specimens are broadened to higher binding energies. The oxygen of the non-biased coating exists in three forms on the sample surface with BE equal to 529.3, 530.1, and 531.6 eV. According to [2], the peak at 529.6 eV corresponds to O 1s in CuO, those located at 530 eV are assigned to O 1s in TiO_2_, while the peak at 531 eV is attributed to the surface adsorbed hydroxide species [52]. The negative shift of 0.2 eV in the main oxygen peak of the biased samples may manifest the non-stoichiometric oxygen atoms because of charge compensation in Cu-O-Ti hybridization [53].

He et al. [54] stated that the peaks at 531.6 and 533.5 eV belong to active surface oxygen species and adsorbed oxygen species where O vacancies are present, respectively. The shift to 0.4 eV lower BE of the active oxygen species compared with the non-biased sample indicates electron transfer between these active species and Ti-Cu-O surfaces [51]. Except for the negative shift, it also found that adding Cu to the −100 V biased sample increases the quantity of chemically adsorbed active oxygen species, which is correlated with an increase in surface oxygen vacancies.

From the high-resolution XPS spectrum of each element, the approximate amount of material on each sample was calculated using the ratio of the integrated peak areas (Table 1). It is worth noting that with the increase in the bias value, the oxygen content rises while the Ti and Cu contents depend on the bias value. The approximate amount of Cu increased at −50 V biasing with a predominant Cu^2+^ state, whereas Ti content decreased. This phenomenon is due to increased ion bombardment when applying a medium bias to the substrates and the attraction and competition between imprisoned atoms to occupy certain lattice sites. It is believed that the sputtering of the film does not occur at zero biasing since the ions have energy below the sputtering threshold. At a negative bias higher than −50 V, the sputtering effect of ions in plasma becomes more effective. The Cu content decreases as the bias voltage rises, as Table 1 illustrates. It is known that copper and titanium have sputtering yields in oxygen that are more than ten times different from one another [55]. During the reverse sputtering at high substrate biasing, copper may undergo higher self-sputtering due to its higher sputtering yield than Ti [56]. This phenomenon, known as “self-sputtering”, etches the coated surface and intensifies with the increase in bias voltage [43]. For this reason, at −100 V bias, the copper amount is lowest, the thickness of the coating reduces (Figure 2), and the Cu^1+^/Cu^2+^ ratio increases due to the high Cu self-sputtering rate. However, in the films deposited at high oxygen partial pressure and high negative bias, the void percentage exhibits an overall growing trend because, in the negatively biased electric field around the substrate, the low-energy negative oxygen ions are unable to reach the substrate table sample, increasing the oxygen vacancies in the films [57].

**Figure 4:**
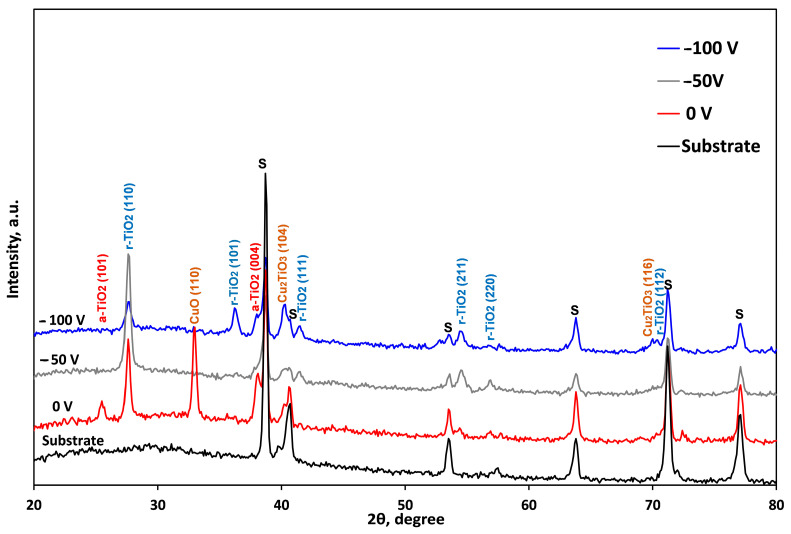
XRD diffraction patterns of the substrate and Cu-doped TiO_2_ coated samples at different bias values.

Figure 4 displays the X-ray diffraction patterns that were obtained experimentally for the bare and coated substrates at various biases. All XRD diffraction maxima are indexed, and no amorphous-like halo can be observed at the lower Bragg angles, meaning that the coatings are crystalline, and no amorphous-like structure is formed in all considered cases. The diffractogram of the non-biased sample shows a mixture of the anatase (a-TiO_2_) and rutile (r-TiO_2_) peaks of TiO_2_ in addition to maxima from the substrate (S). The weight fractions of both rutile and anatase structures as a function of the applied technological conditions of the deposition of the coatings were calculated according to Ref. [58] via relations (2) and (3):

W_rutile_ = I_r_/(K·I_a_ + I_r_) (2)

W_anatase_ = K·I_a_/(I_r_ + K·I_a_) (3)

In Formulas (2) and (3), W_rutile_ and W_anatase_ are the weight fractions of rutile and anatase, respectively; I_r_ and I_a_ are the experimentally obtained intensities of the diffraction peaks corresponding to the rutile and anatase phases, and K represents a coefficient that is equal to 0.886. The results are presented in Table 2.

Based on the results presented in Table 2, the non-biased sample has the highest anatase-to-rutile ratio. At the same time, the weight fraction of anatase is significantly reduced when a medium bias voltage is applied during the deposition process. On the one hand, the increment in the anatase fraction of the biased coatings can be influenced by the higher energetic ion bombardment. When biasing the substrate, the intensity of ion bombardment increases and more energy is transferred to the growing coatings, resulting in higher local temperature and mainly crystalline rutile formation. It was calculated that energy exceeding 7 keV per deposited atom led to the formation of an anatase/rutile mixture with an increasing relative quantity of rutile [59]. This is completely in agreement with the results obtained in the present study. Indeed, rutile crystallises slower than anatase since the latter has lower surface free energy [60]. The (110) plane of the rutile phase showed the strongest diffraction maximum since it is the most closed-packed plane with lower surface free energy [61]. Except for the (110) plane, the (101) plane’s intensity similarly increases at −100 V biasing, suggesting that the greater energy ion bombardment caused the rutile’s micro-volumes to reorient. Defect sites are more likely to occur in the rutile phase because rutile has larger surface free energy than anatase [62], in which defects represent binding sites for copper atoms. 

On the other hand, since the Cu content in the −50 V bias sample is the highest of all samples (Table 1) while the anatase phase in −100 V is higher than in −50 V (Table 2), it follows that the addition of Cu dopant also enhanced the transformation of anatase to rutile. Similar observations were reported by Mungkalasiri et al. for DLI-CVD-produced Cu-TiO_2_ films [63]. However, peaks corresponding to the CuO phase cannot be seen in the biased coatings. One possible explanation for this phenomenon is that the deposited particles have more energy and mobility. Under O-rich conditions, the chemical potential of copper is high enough to form a monoclinic CuO phase with a strong (110) peak in the non-biased sample. With no polarisation, the ion bombardment energy is low, thus the impinging atoms are not able to rearrange themselves [64]. However, the inclusion of copper in the non-polarized sample does not significantly alter “d” interplanar spacing, which is matched with rutile TiO_2_ (d = 0.3247 nm), suggesting that CuO and TiO_2_ remain intact. Under such deposition conditions, the XPS analysis also reveals a higher level of segregation between the phases of copper and titanium oxides. Additionally, following the results of XPS analysis, Cu atoms in the biased samples can occupy the position of Ti^4+^ atoms in the oxide coating to exist in non-fully stoichiometric phases or to form fine nanocrystalline precipitates that are not visible in the XRD analysis. Under substrate biasing, the probability of Cu atoms occupying the vacancies available within the TiO_2_ lattice is much higher and, therefore, the formation of a solid solution of TiO_2_ with some Cu additions can be obtained. The authors of Ref. [65] reported similar outcomes where the whole Cu (17.3 at%) element was dissolved into the TiN lattice, forming a solid solution. In the rutile phase, five oxygen ions are coordinated by two neighbouring titanium ions and copper dopants. It was calculated that when a Ti^4+^ cation is swapped out for a Cu^2+^ cation in the rutile phase, two electrons are removed and two oxygen holes are created [66]. As a result of under-coordination, the titanium ions undergo deformation, moving away from their lattice positions and outside of the vacancy sites. Applying DFT-based studies, Byrne et al. discovered that together with this distortion, the oxygen ion that opposes the vacant space has a shorter Ti-O bond equal to 1.84 Å, whereas the corresponding bonds away from the vacancy site are 2.02 Å [67]. Moreover, after doping, the apical and equatorial Cu-O distances were less than the corresponding Ti-O lengths in undoped TiO_2_. These effects account for the little shortening that was calculated in the interplanar spacing “d” in the biased samples (Table 2), even though Cu has a bigger cation than Ti. 

**Figure 5:**
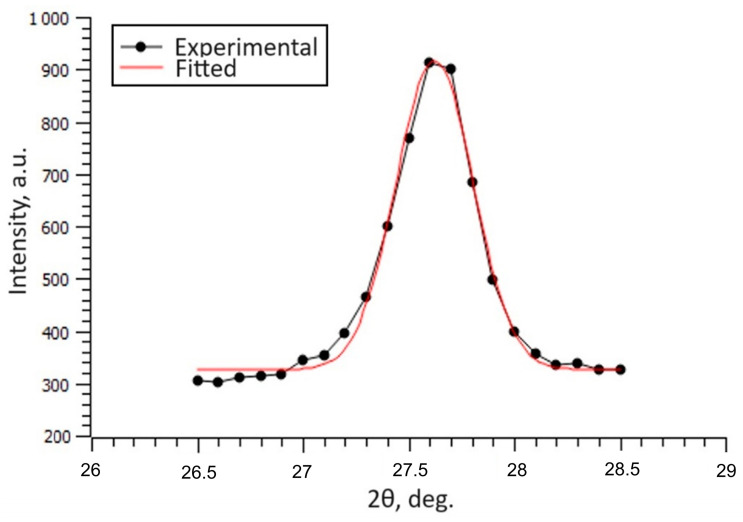
Graphical comparison between the experimental data and fitted (110) peak of the rutile TiO_2_ phase of the non-biased sample.

To analyse the influence of the negative bias voltage applied during the deposition procedure on the crystallographic imperfections (such as dislocations, vacancies, nanopores, stacking faults, etc.), the full width at half maximum (FWHM) of the (110) peak of the rutile phase was measured. Based on the theory of X-ray diffraction, the number of crystallographic imperfections is strongly correlated with the shape of the diffraction maxima, where broader peaks correspond to structures with a higher quantity of defects [68]. Figure 5 shows the accuracy of fitting the (110) peak of the coating deposited without the application of a negative bias voltage in order to confirm the correctness of the determination of the FWHM values. The results show that the FWHM value of the non-biased sample is 0.313 ± 0.012° at 2θ scale (Table 2). The FWHM values increase with the application of −50 V negative bias and rise to 0.428 in the case of the −100 V biased sample. This indicates that more crystallographic imperfections are produced during the deposition of the coatings when a negative bias voltage is used. Additionally, the size of the grains calculated using the well-known Scherrer equation decreases. The increase in the number of crystallographic defects can be associated with the so-called “self-sputtering” effect caused by the ion bombardment as well as by copper doping. 

The experimentally obtained XRD patterns corresponding to the negatively biased samples contain peaks that may be attributed to some composite phases in the Ti-Cu-O system. According to the ICDD (International Center for Diffraction Data, PDF #17-0618) database, these maxima can be associated with the Cu_2_TiO_3_ compound, meaning that not all the Cu was dissolved within the TiO_2_ lattice, and some composite structures were formed in the system of Ti-Cu-O elements. The intensity of the small peaks of Cu_2_TiO_3_ with rhombohedral crystal lattice increased at the highest applied bias. O’Donnell et al. reported that in the copper titanate compound, the oxidation states of copper and titanium are Cu^1+^ and Ti^4+^, respectively [69]. These findings are in good agreement with the XPS data.

**Figure 6:**
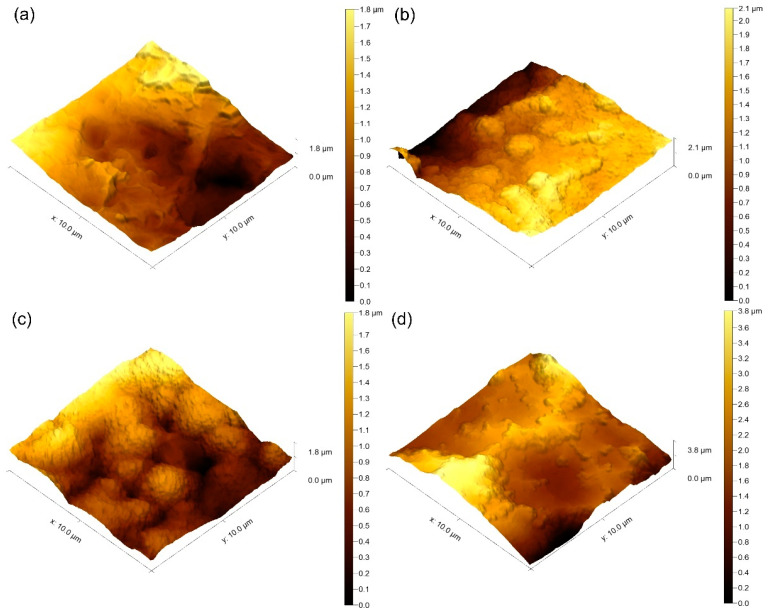
Representative images of surface roughness analysis using AFM (**a**) etched alloy surface; (**b**) Cu-TiO_2_ samples coated at 0 V; (**c**) at −50 V, and (**d**) at −100 V bias voltages.

The chemical composition of the coatings influences the surface wettability, morphology, and roughness [70]. The three-dimensional images obtained by AFM (Figure 6) represent deep valleys and high peaks on the surface of all considered samples. The smallest arithmetical mean (average) roughness (S_a_) and the maximum height of surface (S_z_) values were observed for the 0 V biased sample, while the greatest belonged to the −100 V biased specimen (Table 3). All surfaces are minimally rough (>1 μm) [71] and are supposed to mediate the best combinatory activity of cells involved in bone formation and remodelling around the implant [72]. It can be concluded that with the increase in bias voltage, the roughness of the samples also increases. However, considering the skewness (S_sk_) parameter, which has to do with the asymmetry of the heights’ distribution, it follows that in all samples except −100 V biased ones, the heights of peaks are higher than the depths of the valleys since S_sk_ > 0. On the contrary, the negative S_sk_ value of the −100 V biased sample indicates the dominance of valleys in the profile. The coating etching on the tops under the intense ion bombardment can explain this result.

Indentation tests with 10 mN loadings were conducted on the film surfaces to avoid indenter penetration exceeding 1/10 of the thickness. The hardness and elastic modulus of the substrates and the examined coatings are tabulated in Table 3. It can be seen that the average hardness values of the films increase from about 4.3 GPa up to 15.2 GPa with an increment in deviation values when the bias voltage is increased. There is no relationship between the hardness values and the altered copper content. It follows that the change in hardness of the coatings mainly depends on the microstructure and residual stresses. A looser film structure with reduced hardness forms when no bias is applied because the ions can freely access the substrate surface. The ion bombardment energy rises with the bias voltage, causing the crystal size to decrease (Table 2) and the film density to increase, thus the hardness tends to increase. Moreover, the increase in hardness is also attributed to a reduced anatase-to-rutile ratio since rutile has higher hardness than anatase. For example, magnetron-sputtered rutile film with grains the size of 74 nm demonstrated hardness equivalent to 7.9 GPa (elastic modulus E = 138.5 GPa) whereas an anatase-containing coating with a crystalline size of 33 nm showed a hardness of around 3.5 GPa (E = 115 GPa) [73]. Sputtered and annealed in oxygen, Cu-doped TiO_2_ films with Cu contents from 4.6 to 18 at% demonstrated hardness values from 3.9 to 5.2 GPa [31,32,33,34,35,36,37]. Simultaneously, Cu_2_O films sputtered at different temperatures exhibited hardness values ranging from 7.2 to 12.3 GPa [74]. In our study, besides being deposited in a reactive oxygen atmosphere, the higher hardness values measured for the biased samples can be attributed to the amalgamation of copper and titanium in complex oxide phases with small sizes and a vast number of crystallographic imperfections. Considering the Hall Petch connection, the decrease in grain size causes the boundaries’ area to increase, which delays the dislocations’ sliding and increases surface hardness. The change in the preferential growth of rutile noted in the XRD analysis may also be connected to the rise in hardness of the highest biased coating. Both (110) and (101) planes of rutile TiO_2_ consist of three atomic planes repeated along the direction of stacking [75]. The higher hardness of (101) textured rutile may be because the (101) orientation corresponds to planes of lower atomic packing than the closely packed (110) plane in the tetragonal rutile structure. In addition, the elastic modulus of these films increases when the bias voltage increases because the high energetic particle bombardment causes an increment in the packing density of the films, thus raising the elastic modulus.

**Figure 7:**
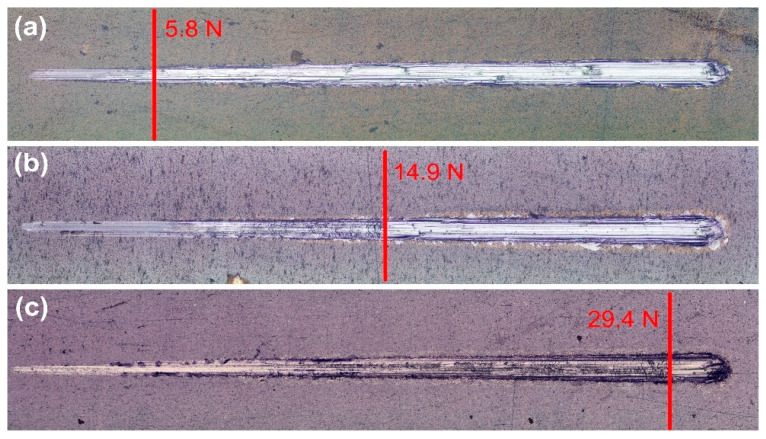
Representative microscopic images of the scratch track on the oxide coatings deposited at (**a**) 0 V, (**b**) −50 V, and (**c**) −100 V bias voltages. The normal load increases from 0 to 30 N from left to right.

Dynamic scratch tests were employed to assess the coating–substrate composite’s load-carrying capability. The critical load values (L_c_) at which the coatings entirely delaminate from the Ti6Al4V substrate were investigated using microscopic observation. Representative images with L_c_ values marked in red are shown in Figure 7. Compared to samples deposited without bias, the biased samples revealed comparatively higher critical loads (Table 3). The main reason is that bias causes the substrate to be bombarded with more energetic particles, which promotes the formation and growth of the pseudo-diffusion transition zone. In contrast to the other samples, small lateral chipping and coating fragmentations were visible along the scratch track of the films with the highest copper content (Figure 7b). The enhanced coating thickness and hardness could be the reason for this phenomenon.

**Figure 8:**
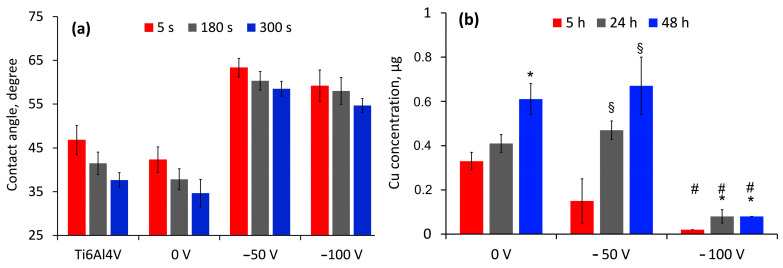
Time evolution of water contact angle (**a**) and release of Cu (**b**) as a function of contact time with 0.9% NaCl at 37 °C of the etched substrate and coated samples at different bias values. * *p* < 0.05 compared to 5 h, § *p* < 0.001 compared to 5 h, # *p* < 0.001 compared to 0 and −50 V for all hours.

The wettability of a surface is associated with spontaneous interaction with the liquid and depends on its surface characteristics such as composition, roughness, topography, etc. The water contact angle (WCA) of all examined surfaces was less than 65 degrees (Figure 8a), meaning that all surfaces are hydrophilic. However, the differences in the WCA values suggest that the form of Cu inclusion in the oxide rather than its content determines the surface wettability. The enhanced hydrophilicity of the 0 V biased sample can be attributed to larger grain sizes and phase separation in contrast to coatings to which a higher bias is applied. The more compact structure of biased coatings increases their surface water contact angle. The slightly lower WCA of the −100 V biased samples is probably due to the higher number of vacancies as opposed to the −50 V biased ones. Garlisi et al. explained this phenomenon by stating that water molecules tend to occupy oxygen vacancies, thus producing a large number of adsorbed OH groups, making the surface more hydrophilic [76]. 

After putting a drop of 0.9% NaCl solution to each sample surface and letting it sit at 37 °C for 5, 24, and 48 h, we measured Cu release using AAS analysis. As shown in Figure 8b, initial copper ion release is higher for the non-biased sample with a greater water contact angle. Though there are differences in the copper content of the 0 V (15.3 at%) and −50 V (20.7 at%) coated samples, copper release from the samples incubated for 24 and 48 h is almost similar (with a statistically insignificant difference). This effect corresponds to the different phase compositions, crystallographic properties of the thin films, and the hydrophilicity of both surfaces. It is well known that the aforementioned structural parameters strongly depend on the applied technological conditions of the film’s deposition. As already mentioned, applying a negative bias voltage to the substrate significantly influences the phase composition of the deposited coatings. The phase composition of the film deposited at a bias of 0 V (unbiased specimen) consists of TiO_2_ (rutile and anatase) and CuO, while the application of a bias of −50 V or −100 V led to the formation of TiO_2_ with dissolved Cu within the matrix. According to the authors of [77], the metal ion release and the corresponding biological and antibacterial properties are very different based on the structure of the film. The complicated mechanism of Cu ions separation in the case of unbiased and −50 V biased specimens could be attributed to the very different phase compositions and crystallographic structures of the coatings. It is suggested that the rate of ion release from solid solutions is different from that where double-phase structures or even more complicated compositions without the dissolution of elements into the main phase are observed [78]. Predictably, the −100 V biased coatings exhibited the lowest copper release across all samples due to the lowest copper content.

**Figure 9:**
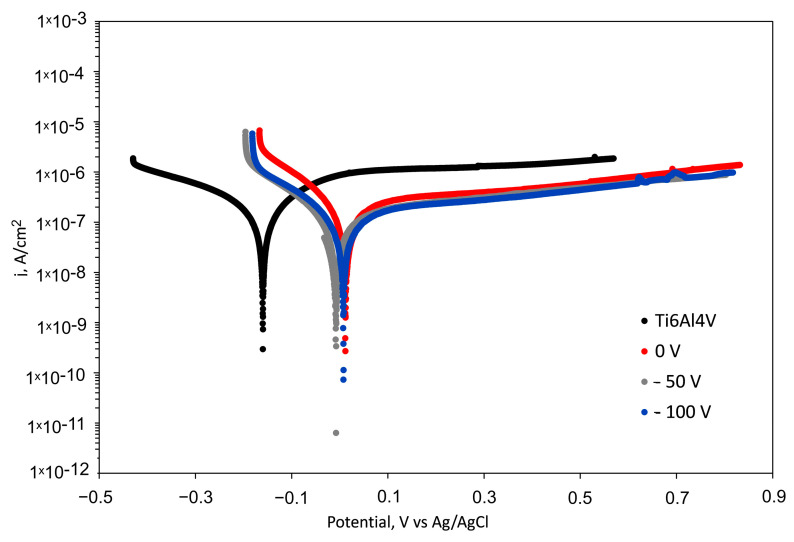
Potentiodynamic polarization curves in SBF solution of the substrate and coated samples at different bias values at 37 °C.

The corrosion properties of the Cu-TiO_2_ coated samples were investigated by utilising electrochemical tests in SBF solution at 37 °C. The results are shown in Figure 9 while Table 4 lists the corrosion potential and corrosion current density values from the polarisation curves using the Tafel extrapolation method. All coated samples indicated a positive shift in E_corr_ and lower corrosion current density than the uncoated alloy. Their passive current density is also lower than that of the bare substrate displaying satisfying pitting corrosion resistance with good passivation protection. E_corr_ values of the coated samples are very close to each other and much higher than that of the bare substrate alloy. Since the E_corr_ value reflects the corrosion tendency of the surface, it follows that it is easier for bare Ti6Al4V to begin corroding. The −50 V biased sample had the lowest j_corr_ value of all samples, indicating a reduced corrosion rate under the particular conditions. This could be because the Cu-doped TiO_2_ coating deposited at −50 V bias forms a denser and thicker film that blocks the diffusion path for the corrosive medium to pass through the defects. Although possessing the lowest content of copper and low passive current density, the −100 V biased sample had a higher j_corr_ than the other coated samples. This fact can be explained by the larger number of defects, the lower thickness, and the higher surface roughness of the −100 V biased sample because of the increased contact area of the film for the solution, which accelerates the possible corrosion processes [79]. The data above indicate that the growing capacity for corrosion resistance is Ti6Al4V < −100 V biased sample < 0 V biased sample < −50 V biased sample.

**Figure 10:**
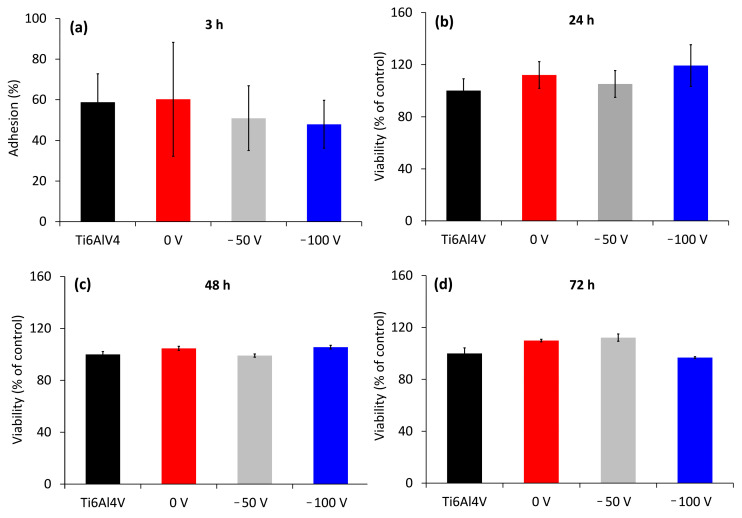
Attachment efficacy of MG-63 osteoblastic cells (**a**) and cell viability after 24 h (**b**), 48 h (**c**), and 72 h (**d**) of MG-63 cell culturing on bare and coated samples at different bias values. Three independent batches of coatings in triplicates were tested. Etched Ti6Al4V was used as a control. The data are shown as average ± standard deviation.

In the first step, we evaluated the adhesion and viability of MG-63 cells by culturing them on both substrate and coated surfaces. The results are illustrated in Figure 10. 

After 3 h of incubation, almost equal numbers of cells were attached to the non-coated and coated samples (Figure 10a). The lack of significant differences suggested good initial cell adhesion on all surfaces studied. After 1 day of cell culturing, the cell viability of Cu-containing samples was slightly higher (Figure 10b). With increasing culture time, MG-63 cells grew similarly on all surfaces, and no significant differences were observed in comparison with etched Ti6Al4V alloy. There were no significant differences in the cell viability of the coated samples and bare control at both 48 and 72 h, indicating that the Cu-containing and etched Ti6Al4V surfaces exhibit similar cytocompatibility. This also implies that the content of Cu ions emitted from the 0 V, −50 V, and −100 V biased films is within the safe range for cells, and the coatings have good in vitro biocompatibility and no significant cytotoxicity. 

**Figure 11:**
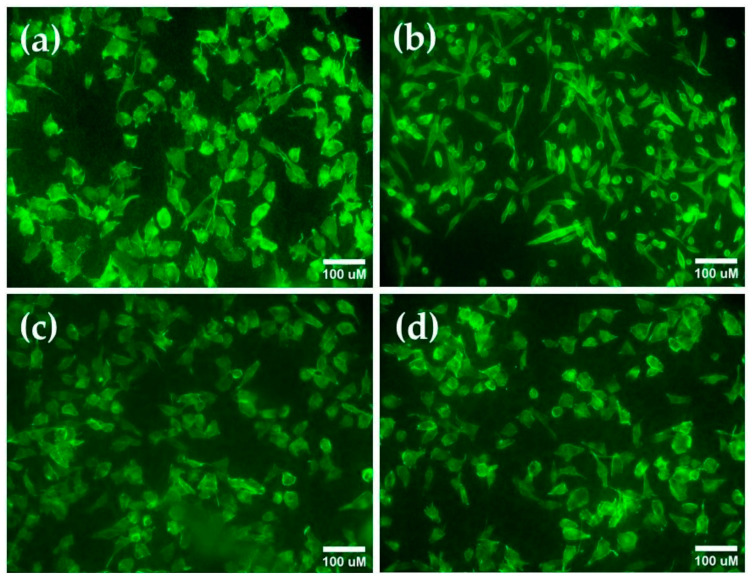
Representative images of F-acting (green) staining of MG-63 osteoblast cells cultured for 24 h on (**a**) etched Ti6Al4V alloy surface and (**b**) Cu-TiO_2_ samples coated at 0 V, (**c**) at −50 V, and (**d**) at −100 V bias voltages. Bar 100 µm.

The next step was to investigate the cell morphology. Figure 11 demonstrates the fluorescence staining images of MG-63 cells cultured on the surfaces for 24 h. In contrast to the better spread polygonal morphology of the cells on the etched Ti6Al4V substrate and −50 V and −100 V biased coatings, those on the 0 V exhibited elongated or spherical shapes (Figure 11b). This observation could indicate that the initial higher copper release after 5 h, the greater hydrophilicity of the sample, and the contact cell surface interactions may have a different effect of 0 V on the spreading of osteoblasts but not on viability (Figure 10b). It is known that Cu^2+^ induces rapid actin polymerization and can cause filament fragmentation [80]. On the other hand, Cu^2+^ ions can increase metallothionein production [81] and tightly control intracellular copper levels to avoid the harmful effects associated with its excess. The spherical shape of cells is an indication of cell division or death. Further investigations are necessary to clarify the impact of the copper released from 0 V biased films on MG-63 cell morphology and metabolism. 

The MG-63 cells cultured on the surfaces of both −50 V and −100 V biased coatings demonstrated cytoskeleton formations similar to the control cell cytoskeleton and spread over the surface. The cytoskeleton assembling speed on the biased coatings could be similar to the non-biased coatings, as evidenced by their polygonal cell shapes with filopodia and lamellipodia extensions. These processes often indicate regular cellular activity and good cell binding to the underlying coatings. All findings suggest that the content of Cu ions emitted from the 0 V, −50 V, and −100 V biased films is within the safe range for cells, thus showing that the investigated coatings have good in vitro biocompatibility and no cytotoxicity.

**Figure 12:**
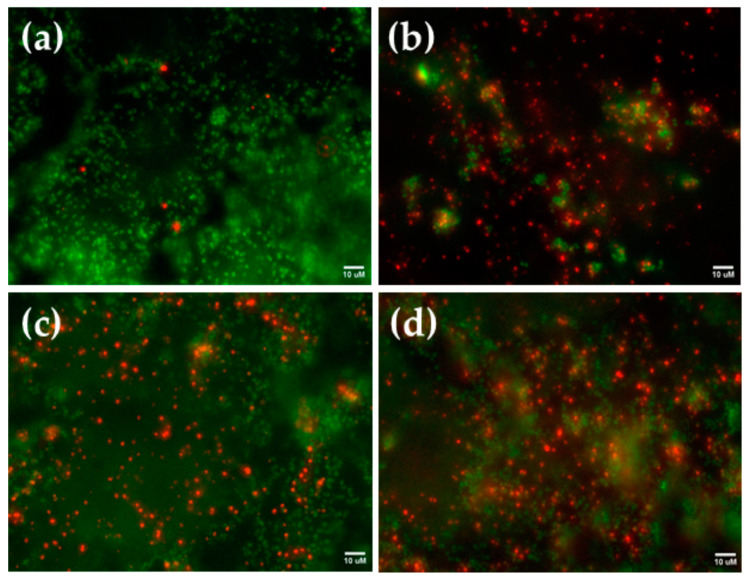
Representative *S. aureus* biofilm formations on the surfaces of (**a**) etched Ti6Al4V alloy; (**b**) samples coated at 0 V, (**c**) −50 V, (**d**) −100 V bias voltages after 24 h incubation. Living bacteria are stained green, while dead bacteria are shown in red.

Following the good cell attachment to the investigated samples, the next question was whether these surfaces had a bactericidal effect. The antibacterial activity of the samples was assessed by comparing two distinct strains of bacteria—*S. aureus* and *E. coli*— which are important colonizers of implants. Biofilm formation was imaged for *S. aureus* and is shown in Figure 12. There were a lot of live bacteria (in green) on the Ti6Al4V alloy and a small number of dead bacteria (in red), implying that the Ti6Al4V alloy surface lacks antibacterial properties. Both dead and living bacteria were present on the −50 V and −100 V coated specimens (Figure 12c,d), confirming similar antibacterial activity by these coatings. The huge bactericidal properties of the 0 V coated sample were demonstrated by the large quantity of red-coloured bacteria on the sample compared to the few green-coloured germs (Figure 12b). 

**Figure 13:**
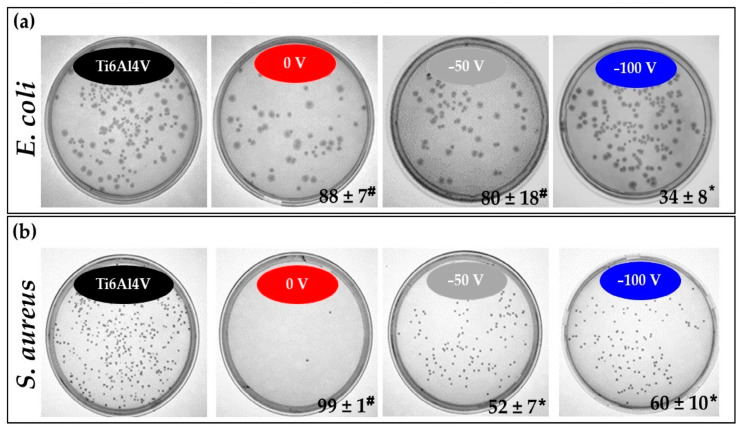
Representative images of (**a**) *E. coli* colonies and (**b**) *S. aureus* colonies on agar plates after 24 h of incubation on the samples’ surfaces. The numbers in the low right corners indicate bacterial growth inhibition versus Ti6Al4V in percentage. The numbers are expressed as mean ± SD (n = 3). The symbols indicate significant differences against control Ti6Al4V. * *p* < 0.033; # *p* < 0.001.

To confirm the results received from biofilm formation, we studied the antibacterial effects of all samples. The results of antibacterial activity against *E. coli* and *S. aureus* colony formation on the different samples are shown in Figure 13. A smaller number of bacterial colonies were seen on all coated samples, in contrast to the numerous colonies on Ti6Al4V, demonstrating that the non-coated alloy possesses no bactericidal activity. The antibacterial properties of the non-biased coating are strongest against both strains. We demonstrated that the interaction between the coating and Cu ion released was sufficient to kill over 88 ± 7% of *E.coli* and 99 ± 1% of *S. aureus* within 24 h. Previous research showed that the anti-microbial activity of copper ions caused an increase in the influx of Cu^2+^ into bacteria and the production of reactive oxygen species and triggered the loss of the integrity of the cytoplasmic membrane, the suppression of respiration, and the destruction of DNA [82]. In our experiments, we observed that *E.coli* and *S. aureus* had different growth patterns when they were cultivated on −50 and −100 V biased coatings. In the case of *E.coli*, the percentage inhibitions of bacterial growth on −50 V and 0 V biased coatings were 80 ± 18% and 88 ± 7%, respectively, and was in full accord with our previous observation that a similar amount of copper ions is released after 24 h. When *E.coli* was cultivated on −100 V biased coatings, the inhibition of bacterial growth was only 34 ± 8%. In the case of *S. aureus*, the inhibition of bacterial growth on −50 V and −100 V biased coatings was similar—52 ± 7% and 60 ± 10%, respectively. Growth inhibition at 0 V biased coating was 99 ± 1% against *S. aureus* as opposed to 88 ± 7% against *E. coli*. The antibacterial activity that was noted against *E. coli* can be likely attributed to the release of copper ions from the coatings and the different structures of the cell walls of Gram-negative bacteria.

Interestingly, there seems to be some optimal concentration of the released copper ions and the layer’s structure at which the most significant antibacterial effect against *S. aureus* is observed. Conversely, lower copper concentrations released from the −50 V and −100 V biased layers within 5 h may trigger adaptive defence mechanisms in *S. aureus*, enabling the bacteria to withstand and adapt to elevated copper levels released later. However, it has also been reported that dissolved copper ions were only partially responsible for the overall cytotoxicity that CuO caused [83]. Direct contact with copper-containing surfaces was thought to trigger alterations in the microbial cell wall and cell membrane [84]. The small WCA of the non-biased coating, which implies a larger contact area of exposure to cells, may be more effective in direct interaction with bacteria. The synergetic effect of Cu released and contact killing could contribute to the excellent bactericidal activity of the non-biased coating for *S. aureus*. The relatively high number of bacteria colonies at −50 and −100 V biased coatings grew, indicating their weaker fighting ability against germs. Since copper ion release from the −50 V biased samples was almost similar to that from the non-biased coating at 24 h, while the highest biased coating possesses the lowest copper content, it appeared that the release of copper ions until 24 h played a significant role in antibacterial effect against *S. aureus* (Figure 13b). Moreover, the WCA of the −50 V biased surface was found to be the highest of all tested samples, indicating a smaller contact area with proteins and cells. Since copper has a greater affinity for proteins than lipids [85], more hydrophobic copper-containing coatings cannot quickly kill Gram-positive bacteria whose cell walls include higher amounts of peptidoglycan and protein.

Bactericidal activity against *E. coli* (Figure 13a) seems more dependent on Cu ion release from the coatings than against *S. aureus*. The Gram-negative *E. coli* bacteria, in contrast to the Gram-positive *S. aureus* species, possess an outer membrane that functions as a permeability barrier, obstructing the passage of biocides into the inner plasma membrane. Numerous investigations have demonstrated that exposure to copper directly targets the cell membrane [86,87], eventually unveiling the cell’s constituent parts, causing a loss of membrane integrity and cell death.

Since the PVD process allows the composition and morphology of Cu-doped TiO_2_ coatings to be controlled, such films can be both non-cytotoxic to osteoblast cells and durably bacteriostatic against *S. aureus* and *E. coli*. Based on the above results, the examined coatings provide enhanced antibacterial activity, protection against aggressive attacks, good cell adhesion, and viability of osteoblastic cells. However, the current study has been performed under in vitro conditions. The copper content in the bone around the implant depends on the tissue’s diffusion rate. More in-depth research on their biological effect and safety is needed.

## 4. Conclusions

The combination of titanium implants’ inherent medicinal qualities with extra antibacterial and bioactive qualities would undoubtedly help to increase their medical efficacy. Achieving the perfect performance in coating development involves striking a balance between superior bio-functionality and potent antibacterial activity. Using a glow-discharge sputtering process to create multifunctional Cu-doped Ti-oxide coatings in a single-step environmentally friendly and scalable procedure is appealing for producing new-generation complex tissue implant devices. The coatings with changing Cu atomic contents from 3 to 20.7 at% indicated copper present in separate phases (CuO) or Cu-dissolved rutile TiO_2_ and ternary oxides. Although containing the highest amount of Cu, the coatings at −50 V bias voltage released similar ions into the solution and had higher hardness, adhesion to the substrate, and roughness values than the non-biased coatings. Moreover, the −50 V biased coatings possess good biocompatibility and are beneficial to cell spreading while displaying good antibacterial activity, especially against *E. coli*. Maintaining comparatively high ion release and more hydrophilic surfaces, the non-biased films showed changed osteoblastic cell morphology and excellent antibacterial properties. Our novel findings in this work demonstrate that reactive co-sputtering enables careful control of the Cu atomic concentration and phase composition of the Cu-doped Ti-oxide coatings, resulting in controllable biological performance in vitro. We show that Cu-doped TiO_2_ films are biocompatible, non-cytotoxic, and have increased surface antimicrobial activity, all of which support the films’ potential as safe and efficient coatings for biomedical applications that can handle the difficulties caused by infectious contamination.

Further research is needed to evaluate the surface’s interactions with bacteria and osteoblast cells. Long-term results and research using co-culture models with different microbiomes should be conducted to validate the findings.

## Figures and Tables

**Table 1:** Average elemental content (in at%) of the coatings determined via XPS analysis.

Sample	O	Cu		Ti	
		Cu^1+^	Cu^2+^	CuTiO_x_	Ti^4+^
Coated 0 V bias	65.9	-	15.3	-	18.8
Coated −50 V bias	64.7	20.7		14.6	
		4.6	16.1	3.3	11.3
Coated −100 V bias	71.7	3.0		25.3	
		1.2	1.8	7.1	18.2

**Table 2:** Calculated parameters of rutile (110) maximum together with anatase and rutile percentages.

Sample	Rutile (110) Maximum			W_rutile_, %	W_anatase_, %
	FWHM, °	d, nm	Grain Size, nm		
0 V	0.313 ± 0.012	0.3226	27.31	58	42
−50 V	0.358 ± 0.011	0.3225	23.88	94	6
−100 V	0.428 ± 0.023	0.3224	19.98	79	21

**Table 3:** Arithmetic mean roughness values (S_a_), skewness (S_sk_), maximum height of surface (S_z_), nano hardness, modulus of elasticity (E), and critical loads (L_c_) (±standard deviations) of the examined substrate and sputtered coatings.

Sample	S_a_ (nm)	S_sk_	S_z_ (μm)	Hardness (GPa)	E (GPa)	L_c_ (N)
Ti6Al4V	352.2 ± 72.0	0.4 ± 0.3	2.3 ± 0.5	4.9 ± 0.9	146.9 ± 14.8	-
Coated 0 V bias	271.9 ± 65.4	0.3 ± 0.8	1.7 ± 0.3	4.3 ± 0.7	140.1 ± 12.7	5.9 ± 0.2
Coated −50 V bias	362.6 ± 84.2	0.3 ± 0.3	2.3 ± 0.4	12.7 ± 2.5	191.2 ± 17.2	14.8 ± 0.9
Coated −100 V bias	548.2 ± 79.2	−0.6 ± 0.6	5.0 ± 1.4	15.2 ± 3.3	211.0 ± 26.7	29.1 ± 1.0

**Table 4:** Corrosion potential (E_corr_) and corrosion current density (j_corr_) values obtained for the substrate and coated samples.

Sample	E_corr_, (mV)	j_corr_ (10^−9^ A/cm^2^)
Ti6Al4V	−160	501
Coated 0 V	11.5	152
Coated −50 V	−7.72	137
Coated −100 V	7.6	309

## Data Availability

The data presented in this study are available on request from the corresponding authors.
